# OSmfs: An Online Interactive Tool to Evaluate Prognostic Markers for Myxofibrosarcoma

**DOI:** 10.3390/genes11121523

**Published:** 2020-12-19

**Authors:** Huimin Li, Longxiang Xie, Qiang Wang, Yifang Dang, Xiaoxiao Sun, Lu Zhang, Yali Han, Zhongyi Yan, Huan Dong, Hong Zheng, Yongqiang Li, Wan Zhu, Xiangqian Guo

**Affiliations:** 1 Cell Signal Transduction Laboratory, Bioinformatics Center, Henan Provincial Engineering Center for Tumor Molecular Medicine, Institute of Biomedical Informatics, School of Medical Sciences, Henan University, Kaifeng 475001, China; lihuimin0202@163.com (H.L.); xielongxiang123@126.com (L.X.); dangyifanga@163.com (Y.D.); sunxiaoxiao1654@163.com (X.S.); zhanglu9128@126.com (L.Z.); hangreen5508@163.com (Y.H.); yanzhongyi2008@163.com (Z.Y.); donghuan8626@163.com (H.D.); medhenu@126.com (H.Z.); liyongqiang@vip.henu.edu.cn (Y.L.); 2 School of Software, Henan University, Kaifeng 475001, China; qiangwang@henu.edu.cn; 3 Department of Anesthesia, Stanford University, Stanford, CA 94305, USA; ms.wanzhu@gmail.com

**Keywords:** Myxofibrosarcoma, OSmfs, tool, prognostic markers

## Abstract

Myxofibrosarcoma is a complex genetic disease with poor prognosis. However, more effective biomarkers that forebode poor prognosis in Myxofibrosarcoma remain to be determined. Herein, utilizing gene expression profiling data and clinical follow-up data of Myxofibrosarcoma cases in three independent cohorts with a total of 128 Myxofibrosarcoma samples from The Cancer Genome Atlas (TCGA) and Gene Expression Omnibus (GEO) databases, we constructed an easy-to-use web tool, named Online consensus Survival analysis for Myxofibrosarcoma (OSmfs) to analyze the prognostic value of certain genes. Through retrieving the database, users generate a Kaplan–Meier plot with log-rank test and hazard ratio (HR) to assess prognostic-related genes or discover novel Myxofibrosarcoma prognostic biomarkers. The effectiveness and availability of OSmfs were validated using genes in ever reports predicting the prognosis of Myxofibrosarcoma patients. Furthermore, utilizing the cox analysis data and transcriptome data establishing OSmfs, seven genes were selected and considered as more potentially prognostic biomarkers through overlapping and ROC analysis. In conclusion, OSmfs is a promising web tool to evaluate the prognostic potency and reliability of genes in Myxofibrosarcoma, which may significantly contribute to the enrichment of novelly potential prognostic biomarkers and therapeutic targets for Myxofibrosarcoma.

## 1. Introduction

Based on the morphologic and clinicopathologic classification criteria established in 2002 and redefined in 2013, the myxoid variant of malignant fibrous histiocytoma (MFH) with a predominant myxoid component (>50%) was renamed as Myxofibrosarcoma (MFS) by World Health Organization (WHO) [1,2]. MFS is a common adult sarcoma with the traits of curvilinear vessels in the variable myxoid stroma and multinodular growth of spindle to polygonal cells [3,4]. MFS tumors harbor highly complex karyotypes, often sharing the aberrations observed in leiomyosarcoma (LMS) and undifferentiated pleomorphic sarcoma (UPS) [5]. The overall recurrence rate of MFS is up to 50–60% [6]. To guide the clinical management of MFS, prognostic biomarkers are essential to be explored and developed.

Previous MFS studies by array comparative genomic hybridization (CGH) have demonstrated that MFS is the most highly complicated sarcoma and exhibits a large degree of molecular heterogeneity. The most common copy number gain/amplification was on 5p, occurring in 60% of MFS [7]. The genes in 5p include S-Phase Kinase Associated Protein 2 (*SKP2*) and α-Methylacyl-CoA Racemase (*AMACR*), both of which have been reported to have a cumulative effect and are potential oncogenes involving MFS tumorigenesis [8,9]. Additionally, overexpression of MET Proto-Oncogene, Receptor Tyrosine Kinase (*MET*), CD109 molecule (*CD109*), and *Ezrin* (*EZR*) have been reported as potential biomarkers for the aggressive behavior of MFS [10]. However, the prognosis biomarker and risk stratification for MFS has been less investigated up to now.

Recently, the employment of public omics data has gradually become an important means to discover new prognostic biomarkers. Xie et al. found that *KRT8* is an importantly potential prognostic biomarker of lung adenocarcinoma according to public omics data [11]. Furthermore, multiple public resources, such as OncoLnc [12], UALCAN [13] and Kaplan–Meier Plotter [14], provide an available interface to explore the association of survival with gene expression using data from the Gene Expression Omnibus (GEO) and The Cancer Genome Atlas (TCGA) database. Our previous studies also depicted web tools to determine the prognostic value of genes in leiomyosarcoma [15] and bladder cancer [16]. However, until now, there is no effective tool to enforce the evaluation of the prognostic value of genes in MFS. Collectively, the aim of this study is to develop a specialized tool, named Online consensus Survival analysis (OSmfs) for MyxoFibroSarcoma, to evaluate the prognostic value of genes in MFS.

## 2. Materials and Methods

### 2.1. Data Collection

There were three independent gene expression datasets with clinical follow-up information of MFS collected from GEO and TCGA by searching with the keywords of “Myxofibrosarcoma” and “survival” or “prognosis”. In total, 128 unique MFS cases were chosen for OSmfs construction; the clinical characteristics of each dataset used in OSmfs were listed and summarized in Table 1. The three independent datasets of TCGA, GSE71118 [17], and GSE72545 [18] from TCGA and GEO databases include 25, 39, and 64 myxofibrosarcoma (MFS) samples, with 7, 10 and 21 death events, respectively. Furthermore, only GSE71118 has 10 metastatic MFS samples. Data in TCGA of MFS come from RNA sequencing; however, GSE71118 and GSE72545 belong to microarrays data. The data from different datasets may possess batch effects. The combined datasets mean that each cohort was divided separately into subgroups (based on high vs. low expression of an inputted gene), which are then pooled for survival analysis.

### 2.2. Design of OSmfs

**Figure 1:**
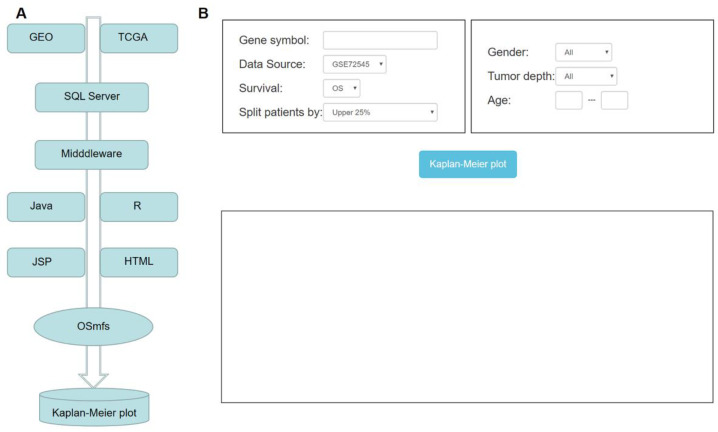
The System flow diagram and operation interface of OSmfs. (**A**) System flow diagram of OSmfs construction. (**B**) The input interface of prognosis analysis in OSmfs.

The method to develop OSmfs has been previously described [15]. In short, the dynamic web interfaces are developed in HTML 5.0 and hosted by Tomcat in a Windows server system. The server-side scripts developed in Java control the output of the analysis results, the R package “R serve,” acts as a middleware to connect R and Java. The SQL Server is used to store and integrate gene expression profiles and clinical data. As the webserver is “out-of-the-box”, when users input the official gene symbol, the prognosis analyses will be performed by the R package “survival” to generate the Kaplan–Meier (KM) curves with a hazard ratio (HR, 95% confidence interval (CI)) and calculate log-rank *p*-value (Figure 1A). OSmfs is freely accessible at http://bioinfo.henu.edu.cn/MFS/MFSList.jsp.

### 2.3. Venny Analysis

Utilizing the data of Cox analysis according to transcription profile data and clinical information in TCGA, GSE71118 and GSE72545, we picked out genes with prognostic significance (*p* < 0.05) in MFS in each of the three datasets, and then compared the three gene lists to look for the common prognostic genes in the three cohorts by Venny 2.0.2.

### 2.4. Receiver Operating Characteristic (ROC) Analysis

Utilizing overall survival (OS) event state (0: Alive; 1: Dead) information, we divided the transcriptional data of one gene into two groups. The area under the curve (AUC) scores represents the capacity for certain genes to predict alive and dead state in overall survival. An AUC of 0.5 represents a test with no discriminating ability, whereas an AUC of 1.0 represents a test with perfect discrimination.

## 3. Results

### 3.1. Application of OSmfs

OSmfs is a web tool that assesses the prognostic value of genes in MFS. To apply OSmfs, users need to input an official gene symbol or a gene signature (one gene per line), specify the “Data Source” including “TCGA”, “GSE71118”, “GSE72545” and “Combined” (the combination of the above three datasets), specify the “Survival” including OS, disease-free interval (DFI), progression-free interval (PFI), progression-free survival (PFS), disease-specific survival (DSS) and metastasis-free survival (*MFS*), and select one cutoff (Upper 25%, Upper 30%, Upper 50%, Upper 25% vs. Lower 25%, Upper 30% vs. Lower 30%, Upper 50% vs. Lower 50%, Lower 25%, Lower 30%, Lower 50%, Trichotomy and Quartile) in “Split patients by” item. With regard to follow-up clinical information, when one selects TCGA or GSE72545 in the “Data Source” column, three clinical factors named Gender (All, Male and Female), Tumor depth (All, Deep, Superficial) and Age (Any scope) can be chosen (Figure 1B) when one selects GSE71118 dataset, clinical factor Metastasis (Metastasis, No and All) can be selected to perform multivariate analysis whose results are illustrated through the Kaplan–Meier curves with HR (95% CI) and log-rank *p*-value, which allow users to evaluate the validity and reliability of prognostic biomarker candidates.

### 3.2. Validation of Prior MFS Biomarkers in OSmfs

To assess the property and reliability of OSmfs, we quarried 12 genes acting as poor prognostic biomarkers in either mRNA or protein level in MFS patients through retrieving Pubmed, then, cleared that the overexpression of 7 genes in the 12 genes, including Integrin Subunit α 10 (*ITGA10*) [18], *CD109* [6], Cyclin Dependent Kinase 6 (*CDK6*) [19], Cyclin Dependent Kinase Inhibitor 2A (*CDKN2A*) [20], *MET* [21], *Cyclin D1* (*CCND1*) [20] and *EZR* [22] predict adverse survival for MFS patients according to OSmfs online analysis (Table 2), however, the other 5 genes including *AMACR* [8], *SKP2* [9], KRAS Proto-Oncogene G TPase (*KRAS*) [23], Epidermal Growth Factor Receptor (*EGFR*) [24], and Argininosuccinate Synthase 1 (*ASS1*) [25] were not identified as prognosticators in MFS possibly due to difference of the size or clinical information of datasets used in previous studies and that of the datasets adopted in OSmfs (Table 2). Moreover, the mRNA-level expression data were utilized in OSmfs while the prognostic role in MFS for genes in ever reports were concluded based on mRNA or protein-level data. Hence, under the absence of prognostic tools for MFS, OSmfs may be an efficient web tool to evaluate the prognostic value of genes and explore new biomarkers in MFS patients.

### 3.3. Identification of Potentially Novel Prognostic Biomarkers in MFS

**Figure 2:**
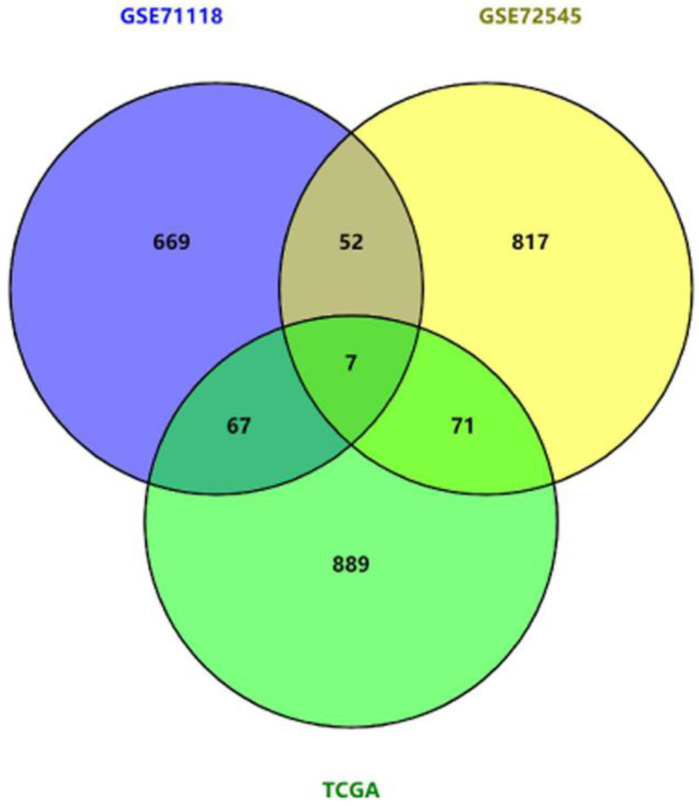
The Venn diagram analysis of the prognostic markers according to Cox analysis (*p* < 0.05) using transcriptome data of MFS from TCGA, GSE71118, GSE72545 cohorts. Seven genes were selected out using the Venny online tool.

To identify novel potential prognostic biomarkers in MFS patients, synthesized cox analysis of patients’ survival was performed with data derived from TCGA, GSE71118 and GSE72545 listed in Table 1. The overlapping results of genes potentially and significantly presenting adverse prognosis illustrated that 7 genes were all associated with poor prognosis of MFS in the three cohorts (Figure 2).

**Figure 3:**
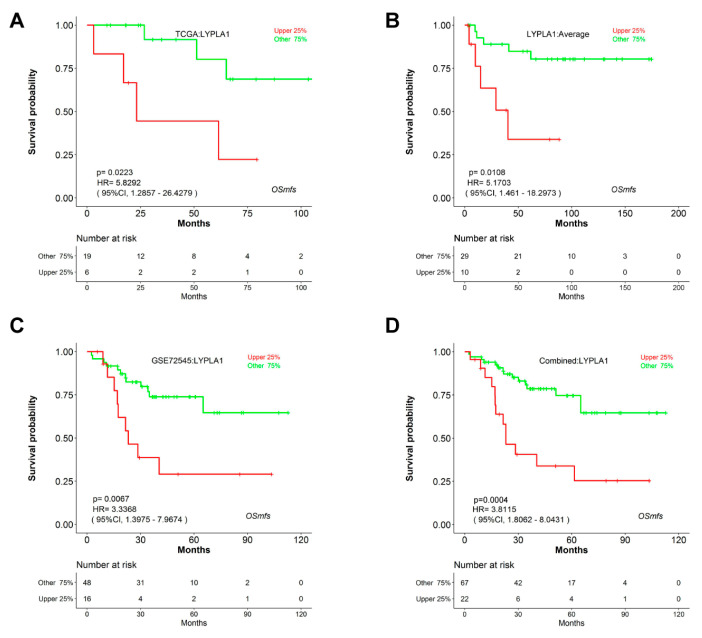
Prognostic analysis of *LYPLA1* in OSmfs. The Kaplan–Meier survival analyses of *LYPLA1* using data in TCGA (**A**), GSE71118 (**B**)**,** GSE72545 (**C**) and Combined (**D**) were shown. *LYPLA1* displays prognostic significance for MFS patients based on survival analysis of the four cohorts.

**Figure 4:**
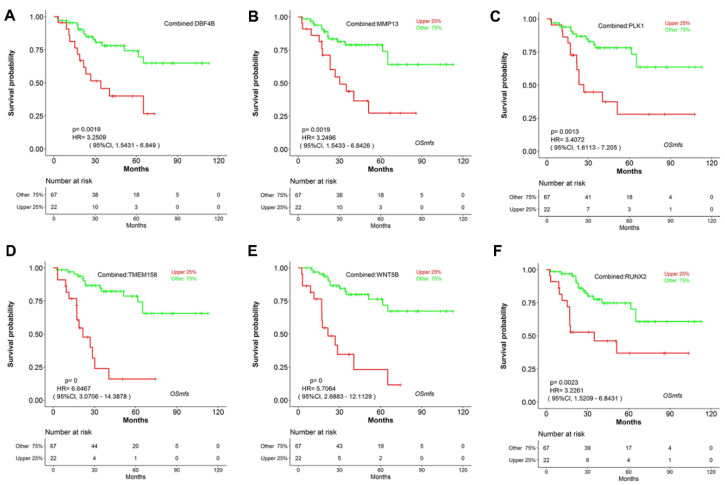
The combined overall survival analysis of *DBF4B* (**A**), *MMP13* (**B**), *PLK1* (**C**), *TMEM158* (**D**), *WNT5B* (**E**), *RUNX2* (**F**) according to TCGA, GSE71118, GSE72545 cohorts in OSmfs.

The gene overexpression of the seven genes named Lysophospholipase 1 (*LYPLA1*), DBF4 Zinc Finger B (*DBF4B*), Matrix Metallopeptidase 13 (*MMP13*), Polo Like Kinase 1 (*PLK1*), Trans- membrane Protein 158 (*TMEM158*), Wnt Family Member 5B (*WNT5B*), and RUNX Family Transcription Factor2 (*RUNX2*) potentially predicted poor overall survival (*p* < 0.05, HR > 1) in MFS based on the three independent cohorts (Table 3). To be specific, *LYPLA1* overexpression independently correlated with worse overall survival (*p* = 0.0223, HR = 5.8292) (Figure 3A) according to TCGA, with adverse metastasis-free survival (*p* = 0.0108, HR = 5.1703) according to GSE71118 (Figure 3B), with worse overall survival (*p* = 0.0067, HR = 3.3368) according to GSE72545 (Figure 3C), and with worse overall survival (*p* = 0.0004, HR = 3.8115) according to combined analysis (Figure 3D), respectively. The role as poor prognostic markers for the other 6 genes including *DBF4B*, *MMP13*, *PLK1*, *TMEM158*, *WNT5B* and *RUNX2* was also reflected using Kaplan–Meier curves in Figure 4A–F. Novelly and in conclusion, in Pubmed searching, none of the 7 genes have been reported to have any association with the prognosis of MFS, suggesting the seven genes may be novelly and potentially prognostic markers in MFS.

**Figure 5:**
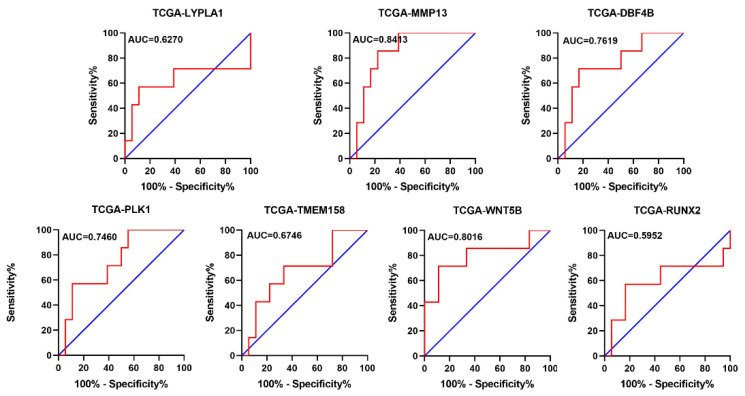
The ROC analyses of overall survival for *LYPLA1*, *MMP13*, *DBF4B*, *PLK1*, *TMEM158*, *WNT5B*, *RUNX2* according to transcription data in TCGA. ROC: receiver operator characteristic, AUC: area under the curve.

**Figure 6:**
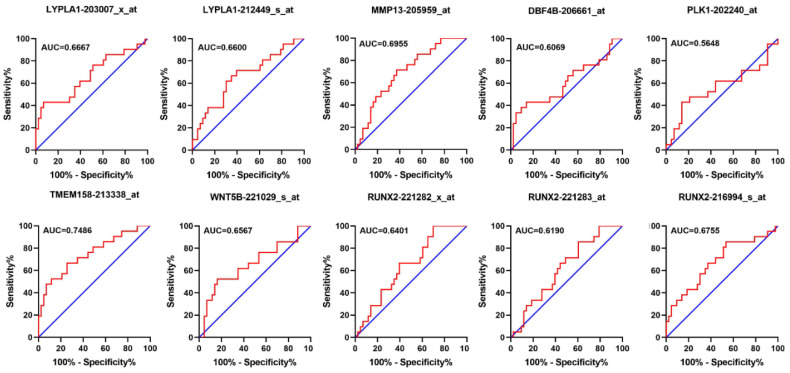
The ROC analyses of overall survival for *LYPLA1* (203007_x_at, 212449_s_at), *MMP13* (205959_at), *DBF4B* (206661_at), *PLK1* (202240_at), *TMEM158* (213338_at), *WNT5B* (221029_s_at), *RUNX2* (221282_x_at, 221283_at, 216994_s_at) according to transcription data in GSE72545. ROC: receiver operator characteristic, AUC: area under the curve.

To further validate the sensitivity and specificity of the 7 genes distinguishing alive and dead state of overall survival in MFS, we performed ROC analyses using transcription data and clinical information in TCGA and GSE72545, the AUC Scores of *LYPLA1*, *MMP13*, *DBF4B*, *PLK1*, *TMEM158*, *WNT5B* and *RUNX2* according to TCGA were 0.6270, 0.8413, 0.7619, 0.7460, 0.6746, 0.8016, 0.5952, respectively (Figure 5), the AUC Scores of LYPLA1 (203007_x_at, 212449_s_at), *MMP13* (205959_at), *DBF4B* (206661_at), *PLK1* (202240_at), *TMEM158* (213338_at), *WNT5B* (221029_s_at), *RUNX2* (221282_x_at, 221283_at, 216994_s_at) according to GSE72545 were respectively 0.6667, 0.6600, 0.6955, 0.6069, 0.5648, 0.7486, 0.6567, 0.6401, 0.6190, 0.6755 (Figure 6), further indicating that the 7 genes may be equipped with the capacity to be prognostic markers.

## 4. Discussion

OSmfs, the first web tool to assess the prognostic potency and reliability of genes in MFS, may be a significant tool for the working scientific community and further contribute to the enrichment of MFS prognostic biomarkers, supplying assistance to doctors or medical oncologists; more exact diagnosis reference needs other validations. To investigate the credibility and specificity of OSmfs, we totally collected 12 genes clarified to possess worse survival in MFS patients. With regard to these markers, the prognostic significance of 7 genes analyzed in OSmfs was consistent with the results ever reported, however, the other 5 genes were not identified as prognosticators in MFS, possibly due to difference in the size or clinical information of datasets used in previous studies and that of the datasets employed in OSmfs. Moreover, the mRNA-level expression data were utilized in OSmfs while the prognostic role in MFS for genes in ever reports were concluded based on protein-level or mRNA data. In addition, the renaming of MFS in 2013 WHO classification which changes the scope of cases designated as MFS, may be also one reason why not all biomarkers candidates previously identified to evaluate patient survival can be verified in our web tool. Interestingly, 3 of the 5 biomarkers unverified in our tool were identified as prognostic biomarkers before 2013. To be highlighted, new potential prognostic biomarkers of MFS patients can also be explored using Cox regression analysis data constructing OSmfs, such as *LYPLA1*, *DBF4B*, *MMP13*, *PLK1*, *TMEM158*, *WNT5B* and *RUNX2*, they were here detected to be potential biomarkers to evaluate poor prognosis for MFS patients. *LYPLA1* acting as a homodimer, exhibits both depalmitoylating as well as lysophospholipase activity, plays a tumor-promotor role in non-small cell lung cancer cells [26]. *DBF4B* is a serine-threonine kinase linking cell cycle regulation to genome duplication. *DBF4B-FL* is required for colon cancer cell proliferation and maintenance of genomic stability [27]. *MMP13* is involved in the breakdown of extracellular matrix in normal physiological processes and disease processes [28], they play a vital role in the prognosis of various cancers such as gastric cancer [29], colorectal cancer [30], and oral squamous cell carcinoma [31]. *PLK1* plays an important role in the initiation, maintenance, and completion of mitosis. Dysfunction of *PLK1* may promote cancerous transformation and drive tumor progression. *PLK1* overexpression was reported to be associated with poor prognoses in a variety of cancers [32]. *TMEM158* facilitates the progression of several carcinomas such as pancreatic cancer [33]. *WNT5B* has been implicated in oncogenesis and developmental processes, including regulation of cell fate and patterning during embryogenesis. Ever report indicated *WNT5B* could serve as a prognostic biomarker in hepatocellular carcinoma [34]. *RUNX2*, a transcription factor, acts as an essential factor in osteoblast differentiation and bone development and regulates a much wider tissue range [35], it could promote breast cancer bone metastasis by increasing integrinα5-mediated colonization [36].

Receiver operating characteristic (ROC) curve analysis acting as an efficient tool is used extensively in medicine to present diagnostic accuracy. Recently, ROC analysis has been commonly used for characterizing the accuracy of medical imaging techniques, non-imaging diagnostic tests, and prediction/risk scores in various settings involving screening, prognosis, diagnosis, staging, and treatment [37]. The area under the ROC curve (AUC) is a global measure of the ability of a test to discriminate whether a specific condition is present or not [38]. ROC curve can also reflect the prognostic ability of the markers. AUC scores in ROC analyses represent the discriminative capacity of the markers [39]. The ROC analyses provide complementary information compared with Cox regression analysis of the potential and novel markers. In this study, the AUC scores which are all greater than 0.5 for the 7 genes indicated the discriminative capacity for the alive and dead state of overall survival in MFS.

## 5. Conclusions

Hence, OSmfs is a potential and significant prognostic analysis tool to evaluate the prognostic value of one gene or a signature in MFS. To be highlighted, the limitations for the tool including that the datasets used to build the tool are small, the datasets come from different platforms and the diagnosis cannot be 100% guaranteed for the included samples are really present. Based on the cancer statistics in 2019, the incidence of sarcomas was not high [40]. Myxofibrosarcoma (MFS) is a unique subtype of soft tissue sarcoma, approximately only 5% of soft tissue sarcoma is diagnosed to be Myxofibrosarcoma [1]. Hence, there are not much public data with regard to Myxofibrosarcoma. To collect as much data as possible, we selected data from different platforms including TCGA and GEO, which may be one reason why the diagnosis cannot be 100% guaranteed for the included samples. OSmfs will be gradually improved and updated to pull in new MFS patients’ data available from TCGA or GEO datasets according to the new criterion defined by WHO in 2013.

## Figures and Tables

**Table 1:** Datasets used in Online consensus Survival analysis for Myxofibrosarcoma (OSmfs).

Dataset	Platform	Clinical Outcomes	No. of Samples	Death Event	Data Sources	Gender (M/F)	Age (Median ± SD)	Metastasis	Tumor Depth (Deep/Superficial)
TCGA	RNAseq	OS, DFI, PFI, DSS, PFS	25	7	TCGA	11/14	60.0 ± 14.78	NA	21/4
GSE71118	GPL570-55999	*MFS*	39	10	GEO	NA	NA	10	NA
GSE72545	GPL96-57554	OS	64	21	GEO	23/41	63.5 ± 16.67	NA	52/12

OS: Overall survival; DFI: Disease free interval; PFI: Progression free interval; DSS: disease specific survival; PFS: progression free survival; *MFS*: Metastasis free survival (The Italic is aimed to differentiate Metastasis free survival and Myxofibrosarcoma); NA: Not available. SD: Standard Deviation.

**Table 2:** Analysis of known prognostic biomarkers in OSmfs.

Literature Results							Validation Results				
Genes	Sample (n)	Detection Level	Clinical Outcomes	Valida-Tion	References	Issuing Time	Clinical Outcomes	HR(95%CI)	*p* Value	Probe ID	Datasets
*ITGA10*	64	RNA	DSS	Yes	18	2016	OS	6.66 (2.76–16.08)	<0.0001 ^a^	206766_at	GSE72545
*CD109*	37	Protein	OS	Yes	6	2015	OS	5.03 (1.11–22.92)	0.0366 ^a^		TCGA
							PFS	4.26 (1.40–12.96)	0.0105 ^c^		TCGA
*CDK6*	77	Protein	*MFS*, DSS	Yes	19	2012	*MFS*	4.14 (1.19–14.44) 5.04 (1.41–17.98)	0.0258 ^b^ 0.0127 ^b^	224847_at 221198_at	GSE71118
*CDKN2A*	116	mRNA protein	OS	Yes	20	2017	OS	2.86 (1.20–6.83)	0.0177 ^a^	211156_at	GSE72545
							*MFS*	3.52 (1.01–12.29) 3.52 (1.01–12.29)	0.0483 ^b^ 0.0483 ^b^	207039_at 209644_x_at	GSE71118
*MET*	86	Protein	*MFS*, OS	Yes	21	2010	OS	5.45 (2.29–12.98) 4.12 (1.73–9.77) 3.36 (1.42–7.95) 5.07 (2.10–12.21)	0.0001 ^a^ 0.0013 ^a^ 0.0057 ^a^ 0.0003 ^a^	203510_at 213816_s_at 211599_x_at 213807_x_at	GSE72545
*CCND1*	116	mRNA	OS	Yes	20	2017	OS	4.00 (1.68–9.54) 4.59 (1.93–10.90)	0.0018 ^a^ 0.0006 ^a^	208712_at 208711_s_at	GSE72545
*EZR*	78	Protein	*MFS*, DSS	Yes	22	2010	OS	10.29 (1.22–86.65)	0.032 ^a^		TCGA
*AMACR*	105	Protein	DSS, *MFS*	Yes	8	2014	*MFS*	1.45 (0.37–5.60)	0.5935	Average	GSE71118
							OS	0.54 (0.21–1.43)	0.2186 ^d^		Combined
*SKP2*	82	mRNA	*MFS*, DSS, OS	Yes	9	2006	*MFS*	2.65 (0.74–9.46)	0.1328	Average	GSE71118
							OS	1.53 (0.71–3.32)	0.2797 ^d^		Combined
*KRAS*	35	mRNA protein	OS	Yes	23	2009	OS	0.54 (0.40–2.21)	0.8897 ^d^		Combined
*EGFR*	47	Protein	OS	Yes	24	2004	OS	1.72 (0.79–3.72)	0.1704 ^d^		Combined
*ASS1*	90	Protein mRNA	DSS, *MFS*	Yes	25	2013	OS	1.50 (0.69–3.25)	0.3086 ^d^		Combined

*ITGA10*: Integrin Subunit α 10; *CD109*: CD109 Molecule; *CDK6*: Cyclin Dependent Dependent Kinase 6; *CDKN2A*: Cyclin Dependent Kinase Inhibitor 2A; *MET*: MET Proto-Oncogene, Receptor Tyrosine Kinase; *CCND1*: Cyclin D1; *EZR*: Ezrin; *AMACR*: α-Methylacyl-CoA Racemase; *SKP2*: S-Phase Kinase Associated Protein 2; *KRAS*: KRAS Proto-Oncogene G TPase; *EGFR*: Epidermal Growth Factor Receptor; *ASS1*: Argininosuccinate Synthase 1. Statistic Significance: *p* < 0.05. ^a^: Significant *p*-value of OS; ^b^: Significant *p*-value of MFS; ^c^: Significant *p*-value of PFS; ^d^: No significant *p*-value validated in any cohorts for OS. OS: overall survival; PFS: progression-free survival; *MFS*: metastasis-free survival; DSS: disease-specific survival.

**Table 3:** The novel biomarker candidates of MFS.

Genes	Data Source	Outcome	*p* Value	HR (95%CI)	Cut-Off
*LYPLA1*	TCGA	OS	0.0223	5.83 (1.29–26.43)	Upper 25%
	GSE71118	MFS	0.0108	5.17 (1.46–18.30)	Upper 25%
	GSE72545	OS	0.0067	3.34 (1.40–7.97)	Upper 25%
*DBF4B*	TCGA	OS	0.0099	7.42 (1.62–34.00)	Upper 25%
	GSE71118	MFS	0.0111	5.18 (1.46–18.47)	Upper 25%
	GSE72545	OS	0.0438	2.44 (1.03–5.79)	Upper 25%
*MMP13*	TCGA	OS	0.0264	5.62 (1.22–25.84)	Upper 25%
	GSE71118	MFS	0.0003	13.13 (3.31–52.13)	Upper 25%
	GSE72545	OS	0.0216	2.76 (1.16–6.55)	Upper 25%
*PLK1*	TCGA	OS	0.0088	19.61 (2.12–181.55)	Upper 25%
	GSE71118	MFS	0.0368	3.77 (1.08–13.14)	Upper 25%
	GSE72545	OS	0.0296	2.62 (1.10–6.25)	Upper 25%
*TMEM158*	TCGA	OS	0.0143	18.57 (1.79–192.47)	Upper 25%
	GSE71118	MFS	0.0293	4.03 (1.15–14.12)	Upper 25%
	GSE72545	OS	<0.0001	5.98 (2.50–14.30)	Upper 25%
*WNT5B*	TCGA	OS	0.0099	7.42 (1.62–34.00)	Upper 25%
	GSE71118	MFS	0.012	5.02 (1.43–17.68)	Upper 25%
	GSE72545	OS	0.0002	5.14 (2.16–12.24)	Upper 25%
*RUNX2*	TCGA	OS	0.024201	8.17 (1.32–50.71)	Upper 25%
	GSE71118	MFS	0.006546	5.92 (1.64–21.36)	Upper 25%
	GSE72545	OS	0.015861	2.92 (1.22–6.97)	Upper 25%

*LYPLA1*: Lysophospholipase 1; *DBF4B*: DBF4 Zinc Finger B; *MMP13*: Matrix Metallopeptidase 13; *PLK1*: Polo Like Kinase 1; *TMEM158*: Transmembrane Protein 158; *WNT5B*: Wnt Family Member 5B; *RUNX2*: RUNX Family Transcription Factor2.

## References

[1] Roland C., Wang W., Lazar A., Torres K. (2016). Myxofibrosarcoma. Surg. Oncol. Clin. N. Am..

[2] Jo V., Fletcher C. (2014). WHO classification of soft tissue tumours: An update based on the 2013 (4th) edition. Pathology.

[3] Haglund K., Raut C., Nascimento A., Wang Q., George S., Baldini E. (2012). Recurrence Patterns and Survival for Patients With Intermediate- and High-Grade Myxofibrosarcoma. Int. J. Radiat. Oncol. Boil. Phys..

[4] Sanfilippo R., Miceli R., Grosso F., Fiore M., Puma E., Pennacchioli E., Barisella M., Sangalli C., Mariani L., Casali P. (2010). Myxofibrosarcoma: Prognostic Factors and Survival in a Series of Patients Treated at a Single Institution. Ann. Surg. Oncol..

[5] Ogura K., Hosoda F., Arai Y., Nakamura H., Hama N., Totoki Y., Yoshida A., Nagai M., Kato M., Arakawa E. (2018). Integrated genetic and epigenetic analysis of myxofibrosarcoma. Nat. Commun..

[6] Emori M., Tsukahara T., Murata K., Sugita S., Sonoda T., Kaya M., Soma T., Sasaki M., Nagoya S., Hasegawa T. (2015). Prognostic impact of CD109 expression in myxofibrosarcoma. J. Surg. Oncol..

[7] Barretina T., Taylor B., Banerji S., Ramos A., Lagos-Quintana M., DeCarolis P., Shah K., Socci N., Weir B., Ho A. (2010). Subtype-specific genomic alterations define new targets for soft-tissue sarcoma therapy. Nat. Genet..

[8] Li C., Fang F., Lan J., Wang J., Kung H., Chen L., Chen T., Li S., Wang Y., Tai H. (2014). AMACR Amplification in Myxofibrosarcomas: A Mechanism of Overexpression That Promotes Cell Proliferation with Therapeutic Relevance. Clin. Cancer Res..

[9] Huang H., Kang H., Li C., Eng H., Chou S., Lin C., Hsiung C. (2006). Skp2 overexpression is highly representative of intrinsic biological aggressiveness and independently associated with poor prognosis in primary localized myxofibrosarcomas. Clin. Cancer Res..

[10] Scoccianti G., Ranucci V., Frenos F., Greto D., Beltrami G., Capanna R., Franchi A. (2016). Soft tissue myxofibrosarcoma: A clinico-pathological analysis of a series of 75 patients with emphasis on the epithelioid variant. J. Surg. Oncol..

[11] Xie L., Guo J., Sun X., Xie T., Zhang L., Yan Z., Amin H., Guo X. (2019). High KRT8 Expression Independently Predicts Poor Prognosis for Lung Adenocarcinoma Patients. Genes.

[12] Anaya J. (2016). OncoLnc: Linking TCGA survival data to mRNAs, miRNAs, and lncRNAs. PeerJ Comput. Sci..

[13] Chandrashekar D., Bashel B., Balasubramanya S., Creighton C., Ponce-Rodriguez I., Chakravarthi B., Varambally S. (2017). UALCAN: A Portal for Facilitating Tumor Subgroup Gene Expression and Survival Analyses. Neoplasia.

[14] Menyhárt O., Nagy Á. (2018). Determining consistent prognostic biomarkers of overall survival and vascular invasion in hepatocellular carcinoma. R. Soc. Open Sci..

[15] Wang Q., Xie L., Dang Y., Sun X., Xie T., Guo J., Han Y., Yan Z., Zhu W., Wang Y. (2019). OSlms: A Web Server to Evaluate the Prognostic Value of Genes in Leiomyosarcoma. Front. Oncol..

[16] Zhang G., Dong H., Yang M., Xie L., Yuan Q., Zhu W., Dang Y., Sun X., Wang Y., Guo X. (2019). OSblca: A Web Server for Investigating Prognostic Biomarkers of Bladder Cancer Patients. Front. Oncol..

[17] Tom L., Gaëlle P., Marine R., Céline B., Pauline L., Valérie D., Carlo L., Agnès N., Philippe T., Dominique V. (2016). RNA sequencing validation of the Complexity INdex in SARComas prognostic signature. Eur. J. Cancer.

[18] Okada T., Lee A., Qin L., Agaram N., Mimae T., Shen Y., O’Connor R., López-Lago M., Craig A., Miller M. (2016). Integrin-α10 Dependency Identifies RAC and RICTOR as Therapeutic Targets in High-Grade Myxofibrosarcoma. Cancer Discov..

[19] Tsai J., Li C., Kao Y., Wang J., Fang F., Wang Y., Wu W., Wu L., Hsing C., Li S. (2012). Recurrent Amplification at 7q21.2 Targets CDK6 Gene in Primary Myxofibrosarcomas and Identifies CDK6 Overexpression as an Independent Adverse Prognosticator. Ann. Surg. Oncol..

[20] Heitzer E., Sunitsch S., Gilg M., Lohberger B., Rinner B., Kashofer K., Stündl N., Ulz P., Szkandera J., Leithner A. (2017). Expanded molecular profiling of myxofibrosarcoma reveals potentially actionable targets. Mod. Pathol..

[21] Lee J., Li C., Fang F., Wang J., Jeng Y., Yu S., Lin Y., Wu J., Tsai J., Li S. (2010). prognostic implication of MET overexpression in myxofibrosarcomas: An integrative array comparative genomic hybridization, real-time quantitative PCR, immunoblotting, and immunohistochemical analysis. Mod. Pathol..

[22] Huang H., Li C., Fang F., Tsai J., Li S., Lee Y., Wei H. (2010). Prognostic Implication of Ezrin Overexpression in Myxofibrosarcomas. Ann. Surg. Oncol..

[23] Willems S., Mohseny A., Balog C., Sewrajsing R., Bruijn I., Knijnenburg J., Cleton-Jansen A., Sciot R., Fletcher C., Deelder A. (2009). Cellular/intramuscular myxoma and grade I myxofibrosarcoma are characterized by distinct genetic alterations and specific composition of their extracellular matrix. J. Cell. Mol. Med..

[24] Sato O., Wada T., Kawai A., Yamaguchi U., Makimoto A., Kokai Y., Yamashita T., Chuman H., Beppu Y., Tani Y. (2005). Expression of epidermal growth factor receptor, ERBB2 andKIT in adult soft tissue sarcomas. Cancer.

[25] Huang H., Wu W., Wang Y., Wang J., Fang F., Tsai J., Li S., Hung H., Yu S., Lan J. (2013). ASS1 as a Novel Tumor Suppressor Gene in Myxofibrosarcomas: Aberrant Loss via Epigenetic DNA Methylation Confers Aggressive Phenotypes, Negative Prognostic Impact, and Therapeutic Relevance. Clin. Cancer Res..

[26] Mohammed A., Zhang C., Zhang S., Shen Q., Li J., Tang Z., Liu H. (2019). Inhibition of cell proliferation and migration in non-small cell lung cancer cells through the suppression of LYPLA1. Oncol. Rep..

[27] Chen L., Luo C., Shen L., Liu Y., Wang Q., Zhang C., Guo R., Zhang Y., Xie Z., Wei N. (2017). SRSF1 Prevents DNA Damage and Promotes Tumorigenesis through Regulation of DBF4B Pre-mRNA Splicing. Cell Rep..

[28] Pittayapruek P., Meephansan J., Prapapan O., Komine M., Ohtsuki M. (2016). Role of Matrix Metalloproteinases in Photoaging and Photocarcinogenesis. Int. J. Mol. Sci..

[29] Ren J., Liu J., Sui X. (2018). Correlation of COX-2 and MMP-13 expressions with gastric cancer and their effects on prognosis. J. BUON Off. J. Balk. Union Oncol..

[30] Huang M., Chang H., Chung F., Yang M., Yang Y., Wang J., Lin S. (2010). MMP13 is a potential prognostic marker for colorectal cancer. Oncol. Rep..

[31] Vincent-Chong V., Salahshourifar I., Karen-Ng L., Siow M., Kallarakkal T., Ramanathan A. (2014). Overexpression of MMP13 is associated with clinical outcomes and poor prognosis in oral squamous cell carcinoma. Sci. World J..

[32] Liu Z., Sun Q., Wang X. (2017). PLK1, A Potential Target for Cancer Therapy. Transl. Oncol..

[33] Fu Y., Yao N., Ding D., Zhang X., Liu H., Ma L., Shi W., Zhu C., Tang L. (2020). TMEM158 promotes pancreatic cancer aggressiveness by activation of TGFβ1 and PI3K/AKT signaling pathway. J. Cell Physiol..

[34] Dong J., Ying L., Shi K. (2019). Expression of the Wnt ligands gene family and its relationship to prognosis in hepatocellular carcinoma. Cancer Cell Int..

[35] Ferrari N., McDonald L., Morris J.S., Cameron E.R., Blyth K. (2013). RUNX2 in mammary gland development and breast cancer. J. Cell Physiol..

[36] Li X., Lu J., Tan C., Wang Q., Feng Y. (2016). RUNX2 promotes breast cancer bone metastasis by increasing integrin α5-mediated colonization. Cancer Lett..

[37] Obuchowski N.A., Bullen J.A. (2018). Receiver operating characteristic (ROC) curves: Review of methods with applications in diagnostic medicine. Phys. Med. Biol..

[38] Zhe H., Jane C., Dawn T. (2017). What is an ROC curve?. Emerg. Med. J..

[39] Combescure C., Thomas V.P., Damien C.W., Jean-Pierre D., Yohann F. (2014). Prognostic ROC curves: A method for representing the overall discriminative capacity of binary markers with right-censored time-to-event endpoints. Epidemiology.

[40] Siegel R.L., Miller K.D., Jemal A. (2019). Cancer statistics. CA A Cancer J. Clin..

